# Keeping an Eye on Myocilin: A Complex Molecule Associated with Primary Open-Angle Glaucoma Susceptibility

**DOI:** 10.3390/molecules16075402

**Published:** 2011-06-27

**Authors:** Farid Menaa, Carolina Ayumi Braghini, Jose Paulo Cabral De Vasconcellos, Bouzid Menaa, Vital Paulino Costa, Eugênio Santana De Figueiredo, Monica Barbosa De Melo

**Affiliations:** 1 Laboratory of Human Genetics, Center for Molecular Biology and Genetic Engineering (CBMEG), University of Campinas (UNICAMP), Campinas-SP 13083-875, Brazil; Email: carolina.ayumi@cbmeg.unicamp.br (C.A.B.); melomb@uol.com.br (M.B.D.M.); 2 Department of Ophthalmology, Faculty of Medical Sciences, University of Campinas (UNICAMP), Campinas-SP 13083-888, Brazil; Email: cabraljp@uol.com.br (J.P.C.D.V.); vp.costa@uol.com.br (V.P.C.); eugsantana@ig.com.br (E.S.D.F.); 3 Department of Chemistry and Nanobiotechnology, Fluorotronics, Inc., San Diego, CA 92081, USA; Email: bouzid.menaa@gmail.com (B.M.)

**Keywords:** glaucoma, blindness, myocilin, gene variants, protein folding, molecular aggregation

## Abstract

*MYOC* encodes a secretary glycoprotein of 504 amino acids named myocilin. *MYOC* is the first gene to be linked to juvenile open-angle glaucoma (JOAG) and some forms of adult-onset primary open-angle glaucoma (POAG). The gene was identified as an up-regulated molecule in cultured trabecular meshwork (TM) cells after treatment with dexamethasone and was originally referred to as trabecular meshwork-inducible glucocorticoid response (*TIGR*). Elevated intraocular pressure (IOP), due to decreased aqueous outflow, is the strongest known risk factor for POAG. Increasing evidence showed that the modulation of the wild-type (wt) myocilin protein expression is not causative of glaucoma while some misfolded and self-assembly aggregates of mutated myocilin may be associated with POAG in related or unrelated populations. The etiology of the disease remains unclear. Consequently, a better understanding of the molecular mechanisms underlyingPOAG is required to obtain early diagnosis, avoid potential disease progression, and develop new therapeutic strategies. In the present study, we review and discuss the most relevant studies regarding structural characterizations, expressions, molecular interactions, putative functions of *MYOC* gene and/or its corresponding protein in POAG etiology.

## 1. Introduction

### 1.1. Physiopathology of POAG

Glaucoma includes a group of eye disorders characterized by progressive cupping of the optic nerve head and visual field loss [1]. In fact, this optic neuropathy includes the (i) loss of neural tissue; (ii) activation of glial cells; (iii) tissue remodeling and change of intra-ocular pressure (IOP) that may lead to apoptosis of the retinal ganglion cells [1,2]. One of the mechanisms involved in apoptosis of the retinal ganglion cells includes posterior bowing of the lamina cribrosa, followed by the blockage of the axonal transport and interruption of the delivery of neurotrophins from the superior colliculus to the retinal ganglion cell body [3]. Untreated glaucoma is the leading cause of irreversible blindness in the world. A model projection revealed that, by 2020, 79.6 million of people worldwide will be affected by glaucoma and 5.9 million of individuals will be concerned by glaucoma-associated bilateral blindness [4].

**Figure 1:**
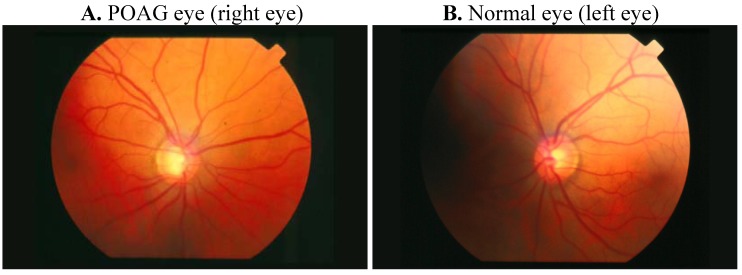
Comparative retinography photograph from the same patient. The optic disc is showed as a bright area where blood vessels converge.

Primary open-angle glaucoma (POAG), also named chronic glaucoma, is the most common form of glaucoma [4]. Unlike angle-closure glaucoma (ACG), *aka* acute or narrow-angle glaucoma, OAG has a wide and open angle between the iris and cornea (Figure 1). There are several risk factors associated with POAG, including demographic, familial, systemic and ocular factors [5]. Both increased age and ancestry are risk factors indentified in transversal and longitudinal studies. The presence of a family history of glaucoma, another well-established risk factor for the development of POAG, has been described among populations of different ethnicities [5]. The early-onset form of POAG, called juvenile open-angle glaucoma (JOAG), frequently shows a Mendelian pattern of inheritance, whereas the most prevalent sub-form, called adult-onset POAG, is inherited as a complex trait in the majority of the cases [6]. Systemic factors, including low perfusion pressure of the optic nerve head, have also been reported in various studies [7]. Finally, increased IOP is a strong risk factor for POAG. Population-based data have shown a positive association between IOP and the incidence of glaucoma [5].

**Figure 2:**
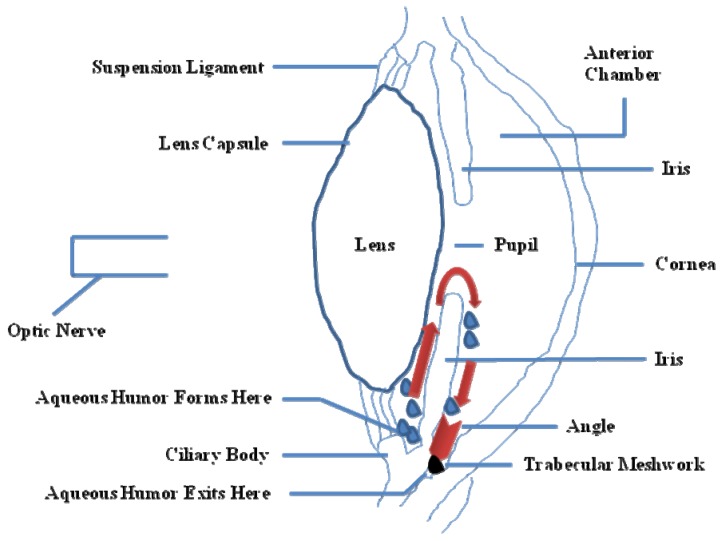
Partial side view of the human eye. Red arrows show the drainage of the aqueous humor from the inner chamber to the Schlemm’s canal at the outer edge of the iris. In an open angle glaucoma (OAG) eye, fluid is unable to exit at the angle and stays within the eye, thus increasing the pressure (IOP).

IOP is essential to keep the shape of the eye, allowing the images to be focused accurately onto the retina, and to maintain an adequate intraocular metabolism. The aqueous humor, produced in the ciliary body, contains oxygen and nutrients to nourish the asnterior segment of the eye. It is drained from the eye into the bloodstream through the sieve-like trabecular meshwork (TM). TM is thought to regulate aqueous humor outflow to control IOP. In brief, TM is composed of cells and matrix and is represented by three structurally different regions: (i) the inner uveal meshwork; (ii) the deeper corneoscleral meshwork; (iii) the juxtacanalicular tissue (*aka* the cribriform meshwork), which is directly adjacent to the inner wall of Schlemm’s canal [8]. In POAG eyes, there is a partial blockage within the TM, especially at the juxtacanalicular tissue, restricting the drainage of aqueous humor and increasing IOP (Figure 2). There is also an increase of the extracellular matrix, and an accumulation of banded fibrillar elements that are embedded in different glycoproteins, denominated “plaque material”, leading to an increase of the trabecular outflow resistance [9].

It has been difficult to find a single marker protein to identify a human TM cell, although the pronounced phagocytosis rate shown by these cells can be used as a specific behavior [10]. One of the most important characteristic of the human TM cell is the increased expression of genes such *MYOC* following exposure to glucocorticoids (e.g., dexamethasone) [10,11,12], or *αB Crystallin*, after transforming growth factor-β (TGF-β), heat shock or oxidative stress treatment [10].

There are usually no symptoms in the initial stages of the glaucomatous disease, but blindness can be prevented if glaucoma is diagnosed and treated early enough. The aim of treatment is to lower the target IOP. Target IOP can be defined as the IOP level where further damage to the optic nerve is likely to be prevented or delayed. Target IOP varies from case to case and depends on age, baseline, untreated IOP, and glaucoma severity [13]. IOP can be lowered by reducing aqueous humor production or by increasing aqueous humor outflow [14]. Indeed, IOP lowering is possible with the use of eye drops, laser treatment directed to TM, which causes ciliary body destruction or, trabeculectomy, a surgical procedure that includes the creation of a guarded fistula to allow communication between the anterior chamber and the subconjunctival space, reducing the resistance to aqueous humor outflow.

Eventually, as suggested from epidemiological and molecular studies, it is now well established that a genetic component may contribute to OAG, and several OAG-associated genes have been identified. The first-identified and the most-studied gene is *MYOC*, encoding for the myocilin protein, which is highly expressed in and secreted by the human TM [12]. Dominant mutations in *MYOC* are more frequently observed in JOAG (10% to 30% of the cases) than in POAG (3% to 4% of patients) [15,16]. Some mutations in the *MYOC* gene lead to the inhibition of mutated myocilin secretion. Secretion of wild-type myocilin (wt myoc) can also be reduced or blocked in the presence of mutated myocilin (mt myoc) [17,18,19]. It has been suggested that the intracellular accumulation of myocilin aggregates is deleterious to the TM cells, resulting in the deterioration of their function and subsequent elevation of IOP [20,21]. In the present study, we review and discuss relevant literature data about the human myocilin molecule with regards to its structure, expression, interactions and potential role(s) in POAG etiology.

### 1.2. Gene Mapping of MYOC

In 1993, Sheffield *et al.* performed a linkage analysis in a five generation family with JOAG using microsatellites as genetic markers. The authors described the first locus associated with OAG, denominated *GLC1A*, mapped in the long arm of chromosome 1 [22]. In 1997, from families affected by autosomal dominant JOAG and POAG, Stone *et al*. identified mutations in the *MYOC* gene, a TM-inducible glucocorticoid response gene (*TIGR*), located in the *GLC1A* interval on chromosome 1q23-q24 [12]. The cDNA was then independently cloned from subtracted ciliary body [23] and retinal cDNA libraries [24].

### 1.3. Expression and Regulation of MYOC

The induction of the *MYOC *gene expression was initially observed in cultured TM cells following treatment with glucocorticoids such as dexamethasone (DEX) [11,25]. It is well known that the long-term use of topical ophthalmic steroids results in IOP with glaucoma, known as steroid-induced glaucoma (SIG) [26]. Interestingly, the profile of *MYOC* up-regulation by DEX was dose- and time-dependent, very similar to the course of development of SIG [25]. Nevertheless, the association between myocilin expression and steroid-induced IOP was not evident [27].

Although *MYOC* gene has been found, by Northern blot, to be expressed as a 2.3 kb transcript in many human tissues (e.g., heart, stomach, thyroid, bone marrow, thymus, prostate, colon) but not in all (e.g., brain, placenta, liver, kidney, spleen, or leukocytes), its highest abundance appeared to be restricted to ocular tissues such as iris, ciliary body, optic nerve, aqueous humor and TM [24,28,29,30,31,32,33,34]. Another study revealed, also by northern blot analysis, that *MYOC* is variably expressed as 2.1 and 1.8 kb transcript isoforms in eye structures [35].

The study of regulatory mechanisms governing glucocorticoid-mediated *MYOC *induction in human TM cells, showed that (i) the promoter region between −2548 and −1541 bp, is required for DEX induction of *MYOC* expression; (ii) *MYOC* is a delayed secondary glucocorticoid-responsive gene; (iii) *MYOC* mRNA is intrinsically quite stable [36]. This *MYOC* mRNA stability can be regulated by over-expression of optineurin, another protein associated with glaucoma [37].

### 1.4. Structural Characterization of MYOC

Genomic sequence analysis revealed that *MYOC *gene is composed of three exons of 604, 126, and 782 bp, respectively [38], which spans 16 kb [39]. Independently, another study confirmed the presence of these exons, and an imperfect palindromic glucocorticoid response element in the 5-prime untranslated region (5’-UTR) was identified [40]. In fact, the 5’-UTR of MYOC gene contains TATA and CAT boxes, MIR and Alu repeat sequences, binding sites for multiple hormone and cell signaling response elements, but it lacks SP1-binding sites [25]. The 3’-UTR contains three polyadenylation signal sequences [35] and not two as previously described in [25]. The encoded protein, myocilin, belongs to a family of glycosylated proteins containing a C-terminal olfactomedin-like (*OLF*) domain [41,42,43]. This domain was originally identified in a glycoprotein isolated from the olfactory epithelium of frogs [44]. This family includes both secreted and membrane-bound proteins with a characteristic distribution in different tissues [45,46,47,48,49,50,51,52,53].

Sequence analysis revealed that human *MYOC* encodes a secreted glycoprotein, myocilin, of a molecular weight of about 55 kDa represented by 504 amino acids, which displays a leucine zipper domain, 10 putative phosphorylation sites and four potential glycosylation sites [38]. Nevertheless, a variant of MYOC with a 338 bp internal deletion removing the coding sequence for the entire leucine zipper region has also been identified in human ocular tissues [35]. Furthermore, it has been observed in aqueous humor and ocular tissues that the human wtmyoc was proteolytically cleaved between arg226 and ile227, resulting in a 35 kDa fragment containing the C-terminal *OLF* domain and a 20 kDa fragment containing the N-terminal leucine zipper domain [54].

**Figure 3:**
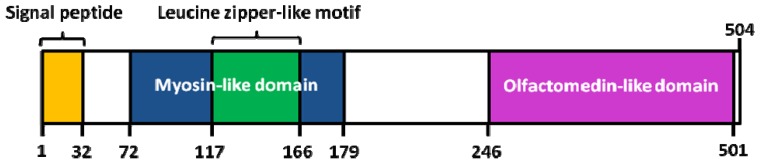
Structure of human myocilin. Colored areas mark the position of the signal peptide, the leucine zipper-like motif and myosin-like domain (N-terminal region), as well as the olfactomedin (*OLF*)-like domain (C-terminal region). Adapted from [55].

Interestingly, a knowledge-based consensus modeling approach [55,56] showed that myocilin is structurally characterized of three main regions (Figure 3): (i) a N-terminal myosin-like coiled-coil region including a leucine-zipper (between amino-acids 111 and 184); (ii) a flexible linker region (between amino-acids 185 and 245); (iii) a C-terminal *OLF* domain (between amino-acids 246 and 504). However, this described model is somehow in partial discordance with a deletion experiment study [57] revealing, a (i) coiled-coil domain located between the amino-acids 78-105; (ii) leucine zipper region between the amino-acids 114-183; (iii) C-terminal domain between the amino-acids 245-504. Functional analysis of myocilin showed that the integrity of amino-terminal coiled-coil regions and olfactomedin homology domain are essential for extracellular adhesion and secretion, the N-terminal region being also important for extracellular interactions (ECM and/or cell surface) [57].

Up-to-date, date the 3D structure of the myocilin is unknown. Indeed, cellular studies have demonstrated temperature-sensitive secretion of myocilin mutants, but difficulties in expression and purification have precluded biophysical characterization of wt myoc and disease-causing mutants *in vitro *[58].

### 1.5. Functions of MYOC

Although the *MYOC* gene has been studied for more than 10 years, the role of myocilin in the POAG etiology is still poorly understood [55,59]. It has been initially speculated that myocilin may cause increased IOP by reduction of the aqueous outflow [12]. Its expression in TM and ciliary body structures involved in the IOP regulation was consistent with this hypothesis [35,37].

Nevertheless, mice with targeted disruption of the *MYOC* gene (Myoc^−/−^) were: (i) viable; (ii) fertile; (iii) without any discernible phenotype; (iv) with a normal IOP, indicating that POAG is not caused by *MYOC* haplo-insufficiency but, might be due to a gain of function [60]. Furthermore, the over-expression of wt myoc to a level similar to that induced by glucocorticoids in the eyes of transgenic mice did not elevate IOP or display any OAG phenotype [61].

Concordantly, a patient examined with a complex deletion of the maternal copy of chromosome 1, including the entire *MYOC* gene, did not present elevated IOP or signs of OAG [62]. Finally, the absence of OAG phenotype in an elderly woman homozygous for the Arg46Stop mutation [63] along with the absence of glaucoma in people hemizygous for *MYOC* [62], suggested that the loss of functional myocilin is not critical for glaucoma development or for normal eye functioning. 

Interestingly, elevated amounts of wt myoc in the aqueous humor of transgenic mice caused significant changes in expression of genes involved in cell adhesion and cell-matrix interactions, supporting a role for myocilin in modulating cellular adhesion [64].

Eventually, it seems that wt myoc alone does not play a major role in the POAG etiology but rather through certain molecular (DNA-protein or protein-protein) interaction that may regulate IOP (see Section 2.1). Most importantly, a glaucoma phenotype appeared to be dependent upon expression and structural conformation of some myocilin mutants in ocular tissues (see Section 2.2).

## 2. Results and Discussion

### 2.1. Myocilin-Molecules Interactions in POAG Etiology

Biochemical and immunoelectron microscopic studies data indicated that myocilin may interact with itself and/or with several intracellular and extracellular matrix (ECM) proteins (e.g., flotillin-1, γ-synuclein, hevin-1, optimedin, GAPDH, fibronectin, fibrillin-1 or type VI collagen) [52,65,66,67,68,69,70,71,72,73], butthe biological significance and clinical relevance of such interactions in the POAG etiology remain unclear. Thereby, myocilin forms *in vivo *(e.g., human aqueous humor, human TM) high molecular weight complexes, ranging from 120 to 180 kDa, due to interaction with itself and other myocilin binding proteins [65,74].

Among the possible molecular interactions that may modulate IOP and thus prevent or contribute to POAG, we can cite: (i) the myocilin-myocilin one, involving the amino-acids 117-166 in the leucine zipper domain, which might be necessary for the myocilin-RLC (myosin regulatory light chain) interaction, suggesting a role for myocilin in the actomyosin system [71,74]; (ii) the interaction of myocilin with components of the Wnt signaling pathways (e.g., Wnt receptors of the Frizzled (Fzd) family, Wnt antagonists of the secreted Frizzled-related protein (sFRP) family and Wnt inhibitory factor 1 (WIF-1)), which modulates the organization of actin cytoskeleton stimulating the formation of stress fibers, critical for the contractility of the TM and IOP regulation [70]. Interestingly, the absence of a glaucoma phenotype resulting from myocilin null mutation (Myoc^−/−^) in the eye could also be explained by the compensatory action of Wnt proteins [70]; (iii) the interaction between the myocilin variant (-1000G) and the apolipoprotein (APOE) variant (-491T) previously associated with Alzheimer disease (AD). Indeed, this interaction involving a single nucleotide polymorphism (SNP) in the respective promoter is associated with increasing IOP and limited effectiveness of IOP-lowering treatments in patients with POAG [72].

A number of genes regulated by glucocorticoids in ocular tissues, or those for which the expression is specifically modulated in POAG tissues, might represent potential myocilin-interacting candidates that may contribute to the progression of glaucoma (data not shown). Indeed, in addition to *MYOC*, other genes were found up-regulated in the DEX-induced human TM [75]. For instance, we can cite those coding the serine protease inhibitors α1-antichymotrypsin and pigment epithelium-derived factor (PEDF), the gene coding the interleukin-8 (IL-8), chemokine involved in cell motility or, the gene coding the carbonic anhydrase XII (CA12), implicated in aqueous humor formation and calcification [74]. Furthermore, comparative analysis of human genome-wide gene expression profiles in POAG TM tissues indicated significant expression changes in genes associated with inflammation (e.g., up-regulation of selectin-E) or anti-oxidation (e.g., down-regulation of paraoxonase 3 and ceruloplasmin) [76].

Eventually, extensive identification and characterization of myocilin-interacting proteins by methods such ChIP-on-chip and/or yeast-two hybrid system may shed light the structure and function of myocilin in the TM and aqueous outflow pathway, thus define the functional protein complex(es) surrounding myocilin protein (wild-type or mutated) implicated in the glaucomatous disease.

### 2.2. MYOC Variants in POAG Etiology

The initial step of POAG pathogenesis may depend upon expression of abnormal mutant myocilin protein, which accumulates into the cell instead of being normally secreted [61,77,78]. A functional assay consisting in transfecting mammalian cells with *MYOC* constructs containing sequence changes seen in glaucoma patients has demonstrated the function of *MYOC *mutations or polymorphisms in the glaucoma pathogenesis [79]. Indeed, the mutants rendered the expressed protein insoluble in the detergent Triton X-100 and would interfere with secretion, dimerization, or interaction of myocilin with ECM components of the TM [79].

Several mutations in *MYOC* gene are continuously described in families with POAG from different ethnical or geographic origins, and mutants databases can freely be accessed online [80]. From those databases, we could notice that the most encountered types of mutations are missense (~86%) or nonsense (~5.5%).

*MYOC* mutations account for about 2%–4% of the POAG patients and, for about 10%–30% of JOAG patients [15,16]. The majority of mutations (over 90%) are concentrated in the evolutionarily conserved C-terminal *OLF* domain encoded by a single exon [81], while other mutations and SNPs have been identified in the promoter, upstream region of the *MYOC* gene [82,83,84,85]. The frequencies of these mutations are usually higher in family studies than in unrelated ones [86].

*In vitro* and *in vivo* studies showed that several *OLF* domain mutations prevented myocilin secretion in physiological temperature conditions, but when cells were cultured at 30 °C, a process known to facilitate protein folding, some sequestered mutants were released in the extracellular medium [57].

One of the major studies carried out in glaucoma patient populations was performed by screening 1703 patients with POAG from five different populations representing three racial groups [87]. There were 1284 patients from Caucasian populations in Iowa (727), Australia (390) and Canada (167) in addition to 312 African American patients from New York City and 107 Asian patients from Japan. Overall, 61 different *MYOC* sequence variations were identified, 21 of which were judged to be probable disease-causing mutations. Among the 21 mutations, 16 (76%) were found in only one population. The most common mutation observed, Q368X, was observed in 27 of the 1703 (1.6%) glaucoma pro-bands and, at least once, in all groups except the Japanese one [87].

We have identified in a Brazilian family pedigree, by single-strand conformation polymorphism (SSCP) and sequencing analyses, the C433R mutation in the *MYOC* gene, which was strictly associated with JOAG/POAG [88]. The penetrance of this mutation was 0% in persons younger than 10 years (0/4), 40% in those between 11 and 30 years (2/5), 75% in those aged from 30 to 40 years (3/4), and 100% in those older than 40 years (4/4) [88].

Interestingly, mutations in both *MYOC* and *CYP1B1 *genes, the later is the most important gene associated with primary congenital glaucoma, were described in a Canadian family segregating both autosomal dominant adult and juvenile onset-POAG [89]. *CYP1B1* encodes a member of the cytochrome P450 superfamily and is co-expressed with *MYOC* in the iris TM, and ciliary body of the eye [89]. All affected family members carried the *MYOC* mutation but those who also had the *CYP1B1* mutation had juvenile onset-POAG, whereas those with only the *MYOC* mutation had the adult-onset form [89]. The mean age at onset of disease among carriers of the *MYOC* mutation alone was 51 years, whereas those of both *MYOC* and *CYP1B1* mutations had an average age of only 27 years [89]. Individuals carrying only a *CYP1B1* mutation were not clinically affected, so it has been concluded that in this family, *CYP1B1* could act as a modifier of *MYOC *[89].

### 2.3. Misfolding and Aggregation of Myocilin in POAG Etiology

Mutations in proteins that induce misfolding and proteasomal degradation are common causes of inherited diseases [90]. It has been found that POAG-causing myocilin mutants were misfolded, highly aggregation-prone, accumulated in large aggregates in the rough ER of human differentiated primary TM cells [21] and formed typical Russel bodies [91].

In TM cells, P370L mutant myocilin, which causes the most severe glaucoma phenotype, was not secreted under normal culture conditions (37 °C), and prolonged expression resulted in abnormal cell morphology and cell killing [21]. However, culture of TM cells at 30 °C facilitated myocilin folding, promoted secretion of mutant myocilin, normalized cell morphology and, reversed cell lethality [21]. ER stress-induced apoptosis is a pathway to explain the reduction of TM cells in patients with myocilin-caused glaucoma [91]. Indeed, the presence of myocilin aggregates induced the unfolded protein response proteins BiP and phosphorylated ER-localized eukaryotic initiation factor-2alpha kinase (PERK) with the subsequent activation of caspases 12 and 3 and expression of C/EBP homologous protein (CHOP)/GADD153, leading to apoptosis [91].

From these observations, myocilin-associated POAG can be considered as an ER storage disease, consisting in a progression of events that involves chronic expression of misfolded and non-secreted myocilin, subsequent TM cell death, TM dysfunction and impediment of aqueous humor outflow leading to elevated IOP [21,91].

In accordance with this observation, several glaucoma-associated *MYOC* mutations, including the P370L one, inhibited calpain II dependent-endoproteolytic processing of full-length myocilin, normally releasing two fragments of ~20 kDa (N-terminal part) and ~35 kDa (C-terminal part), which resulted in accumulation of insoluble mutant myocilin aggregates in the ER [54]. This cleavage might regulate extracellular and matricellular protein interactions (e.g., myocilin-hevin) [67,92], contributing to the control of IOP [93], notably by decreasing myocilin homo-aggregates [94].

In cell culture, the toxicity of mutant myocilins can be reduced by the addition of certain chemical chaperones (e.g., 4-phenylbutyric acid (4-PBA)) or osmolytes (e.g., trimethylamine *N*-oxide (TMAO)) [90,95], and *in vitro*, the compromised stability of myocilin mutants can be restored with some of the same compounds [58]. Indeed, treatment with 4-PBA of cells reduced the amount of insoluble myocilin aggregates and restored the secretion of mutant myocilin [96]. The same treatment in cells co-expressing wt and mt myocilin relieved ER stress and significantly reduced the rate of apoptosis [96]. The ways that a chaperone may help trafficked proteins, both wt and mt, meet the ER quality control requirements, include both thermodynamic- and kinetics-based mechanisms [58]. For instance, the chaperone could (i) accelerate the protein folding; (ii) bind and stabilize a fully folded protein; (iii) stabilize the protein for post-translational modification or interaction with a binding partner required for proper trafficking; (iv) all or some combination of the above [58].

In this context, the protein folding, misfolding and aggregation processes represent a continuous and necessary challenge to better understand the protein function in a given pathology. Unfortunately, the main factors influencing those processes, with regard to myocilin, remain poorly understood. In a general way, the protein structure not only depends on the amino-acid sequence but also on its micro-local environment which enhances the protein helicity [97,98,99]. An efficient *in vitro* strategy to mimic the native *in vivo* protein structure and determine the influencing parameters of protein folding and aggregation, can be obtained by protein encapsulation using an established non-destructive modified sol-gel glass method [97,99]. Indeed, optically transparent biomaterials can facilitate the determination of the protein structure as well as the molar protein ellipticity, stability and biological activity using spectroscopic techniques, such as circular dichroism spectroscopy (CDS) [98]. Interestingly, the first detailed biophysical characterization of *MYOC*-*OLF* domain in solution, to gain insight into its structure and function, has been recently reported [100]. Thereby, it has been shown that *MYOC*-*OLF* is stable in the presence of glycosaminoglycans (GAGs), as well as in a wide pH range in buffers with functional groups reminiscent of such GAGs [100]. CDS analysis of the *OLF* domain revealed significant β-sheet and β-turn secondary structure [100]. At neutral pH, intrinsic tryptophan fluorescence and CDS melts indicated a highly cooperative transition with a melting temperature of ~55 °C. Limited proteolysis combined with mass spectrometry (MS) revealed that the compact core structural of *OLF* domain consists of residues located from position 238 to 461 [100].

### 2.4. Myocilin Is Phylogenetically Well Conserved

**Figure 4:**
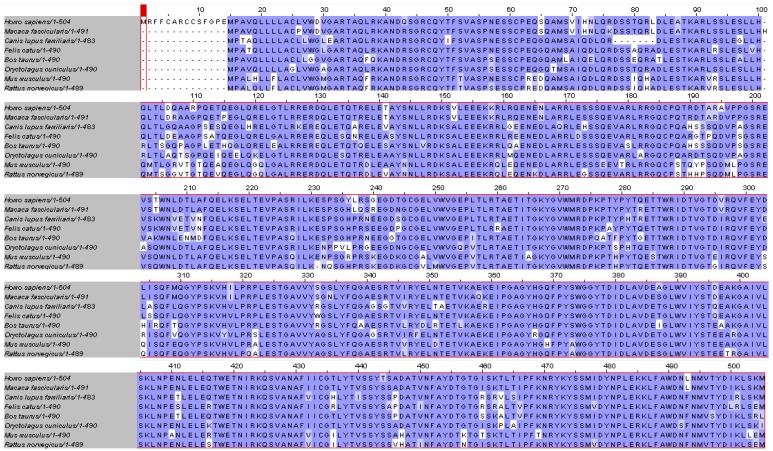
Primary sequence alignment of myocilin from different mammalian species. Protein sequence conservation appears in blue.

Pearson’s sequence format along with the multiple sequence alignment software *ClustalW2 2.0.12* accessible online [101,102] were used to compare the full-length of myocilin primary sequence of different species (n = 8) (Figure 4). First, very few gaps were noticed in the myocilin sequences, and the most important one was observed at the very beginning of the N-terminal sequence where 14 amino-acids are missing in seven species excepted in human (Figure 4). This short amino-acid sequence corresponds to the peptide signal (Figure 3), suggesting human specificity in relation to the myocilin protein cellular trafficking (data not shown). Second, the length of the myocilin protein sequence varied from 483 (*i.e.*, *Canis lupus familiaris*) to 504 amino-acids (*i.e.*, *Homo sapiens*) with an average of 491 amino-acids. Finally, pairwise alignments showed the best score identity between *Homo sapiens* and *Macaca fascicularis* (~96%), while the lower score was observed between *Mus musculus* and *Bos taurus* (~78%). We found that the overall average score of identity, between the eight myocilin sequences was around 77%, demonstrating that myocilin is phylogenetically well conserved. 

To estimate the average distance between the eight myocilin sequences and design a phylogram, we used *BLOSUM62* program integrated to the *ClustalW2 *software.

**Figure 5:**
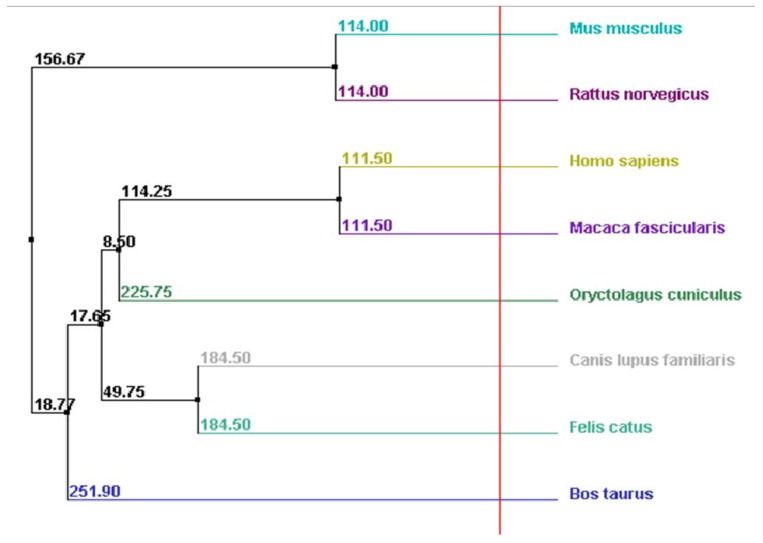
Phylogenic tree showing average distance between mammalian myocilin primary sequences (arbitrary units).

We observed that the most related mammalian species to *Homo sapiens* are respectively *Macaca fascicularis*, *Mus musculus*, *Rattus norvegicus*, *Canis lupus familiaris*, *Felis catus*, *Oryctolagus cuiniculus* and *Bos Taurus* (Figure 5).

### 2.5. Putative 3D-Structure of the Myocilin

**Figure 6:**
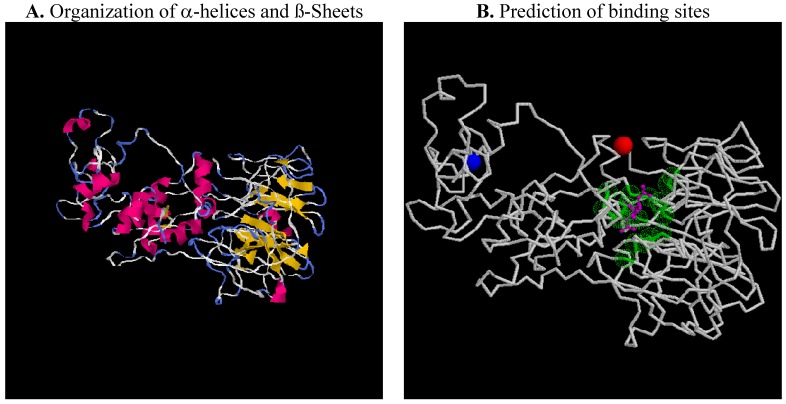
Putative 3D-structure of human myocilin.

One of the possible approaches to determine myocilin 3D protein structure and predict its functions could be the use of the *I-TASSER* program [103,104,105]. Briefly, *I-TASSER* uses state-of-the-art algorithms and generates full length model of proteins by excising continuous fragments from threading alignments, before reassembling them using replica-exchanged *Monte Carlo* simulations. 3D models were built based on multiple-threading alignments by *LOMETS* and iterative *TASSER* simulations. Function insights were then derived by matching the predicted models with protein function databases such “protein databank (PDB)”, available online [106,107]. The most confident predicted protein model in terms of quality/accuracy—based on C-score, TM-score and RMSD of the program—that we found is related to the tRNA wybutosine synthesizing enzyme (TYW4) [108]. TYW4 is an *S*-adenosylmethionine (SAM)-dependent enzyme that catalyzes the final step of wybutosine biosynthesis, methylation and methoxycarbonylation. Based on our preliminary structural studies (Figure 6), we thus suggest that myocilin may be involved in a methylation process.

## 3. Conclusions

POAG is an optic neuropathy with high worldwide prevalence and strong evidence of complex inheritance. Although the POAG etiology remains unclear and may involve several genes associated with the disease, it is now relatively well documented that mutations, mostly encountered in *OLF* domain of the *MYOC* gene, play a critical role in the manifestation of the disease. One molecular mechanism explaining the possible association of some *MYOC* mutations and POAG involves the misfolding and aggregation processes of myocilin protein. Indeed, mutated myocilin can accumulate into the ER of TM, form aggregates, suppress the secretion of normal myocilin and blocks the TM in the eye, which eventually cause cell dysfunction and death, increases the IOP, and eventually leads to POAG. Thereby, myocilin P370L mutant would represent, up-to-date, the one that causes the most severe glaucoma phenotype by eliciting the most potent inhibition of the myocilin processing.

Future challenges may include: (i) genome-wide association studies (GWAS) in order to identify new important submicroscopic genomic alterations such as single-nucleotide polymorphisms (SNPs) but also copy number variations (CNVs) (e.g., deletions, duplications) with the final aim to find causative genes involved in glaucoma, and so better understand the etiology of POAG; (ii) further physical and functional characterization of wt myoc *versus* mutant mt myoc in different micro-environmental conditions; (iii) characterization of dynamic molecular interactions between myocilin-proteins complexes; (iv) unravelling of the 2D/3D myocilin structure (wt *versus* mt myoc); (v) continuous determination of myocilin variants pattern, clinically relevant among worldwide patients populations, in order to establish groups at risk for POAG and allow early diagnosis; (vi) efficient therapy-based control of the IOP–without major post-treatments complications–to avoid potential disease progression.
